# Causes of DNA mismatch repair deficiency in sebaceous skin lesions demonstrating loss of MLH1 protein expression: constitutional over somatic *MLH1* promoter methylation

**DOI:** 10.1007/s10689-025-00456-w

**Published:** 2025-04-10

**Authors:** Jihoon E. Joo, Khalid Mahmood, Mark Clendenning, Romy Walker, Peter Georgeson, Julia Como, Mark A. Jenkins, Michael D. Walsh, Ingrid M. Winship, Daniel D. Buchanan

**Affiliations:** 1 Colorectal Oncogenomics Group, Department of Clinical Pathology, Melbourne Medical School, The University of Melbourne, Parkville, Australia; 2 Collaborative Centre for Genomic Cancer Medicine, Victorian Comprehensive Cancer Centre, The University of Melbourne, Parkville, Australia; 3 Melbourne Bioinformatics, The University of Melbourne, Carlton, Australia; 4 Centre for Epidemiology and Biostatistics, Melbourne School of Population and Global Health, The University of Melbourne, Carlton, Australia; 5 Department of Histopathology, Sullivan Nicolaides Pathology, Bowen Hills, Australia; 6 Department of Medicine, The University of Melbourne, Parkville, Australia; 7 Genomic Medicine and Family Cancer Clinic, Royal Melbourne Hospital, Parkville, Australia

**Keywords:** Sebaceous neoplasia, MLH1 methylation, Constitutional MLH1 epimutation, Lynch syndrome, Muir-Torre syndrome, Mismatch repair-deficiency

## Abstract

Approximately 30% of sebaceous skin lesions (or sebaceous neoplasia) demonstrate DNA mismatch repair (MMR)-deficiency. MMR-deficiency can be caused by Lynch syndrome, resulting from germline pathogenic variants in the DNA MMR genes *MLH1*, *MSH2*, *MSH6* and *PMS2*, but other causes include somatic *MLH1* gene promoter hypermethylation, constitutional *MLH1* gene promoter hypermethylation (*MLH1* epimutation), or biallelic somatic MMR gene mutations. In colorectal (CRCs) and endometrial cancers (ECs), tumour MMR-deficiency showing loss of MLH1 and PMS2 protein expression (MLH1/PMS2-deficiency) is predominantly caused by somatic *MLH1* hypermethylation, however, it is not clear if somatic *MLH1* hypermethylation is a cause of MLH1/PMS2-deficiency in sebaceous neoplasia. This study investigated the causes of MLH1/PMS2-deficiency in 28 cases with sebaceous neoplasia. Germline pathogenic variants in *MLH1* were identified in 11 of 28 cases. Of the remaining 17 non-Lynch syndrome cases, two (11.8%) were positive for *MLH1* hypermethylation in blood-derived DNA (constitutional *MLH1* epimutations). The corresponding sebaceous tissue of these two cases also showed *MLH1* hypermethylation. None of the other eight cases with sufficient sebaceous tissue-derived DNA for testing showed somatic *MLH1* hypermethylation. Multi-gene panel testing of sebaceous tissue and matched blood-derived DNA identified four cases with biallelic somatic *MLH1* mutations as the cause of MLH1/PMS2-deficiency. No cause of MLH1/PMS2-deficiency could be identified in one case. This study demonstrates that biallelic somatic *MLH1* mutations and constitutional *MLH1* epimutations underlie MLH1/PMS2-deficiency in sebaceous neoplasms after excluding Lynch syndrome. Unlike CRCs and ECs, somatic *MLH1* hypermethylation was not identified suggesting it is not a common cause of MLH1/PMS2-deficiency in sebaceous neoplasia.

**Supplementary Information:**

The online version contains supplementary material available at 10.1007/s10689-025-00456-w.

## Introduction

Sebaceous neoplasia refers to a group of skin tumours comprising sebaceous carcinoma, sebaceous adenoma and sebaceoma that originate from oil-producing skin glands, namely the sebaceous glands. Lynch syndrome (LS) is caused by germline pathogenic variants in the DNA mismatch repair (MMR) genes *MLH1*, *MSH2*, *MSH6*, and *PMS2*. People with Lynch syndrome have an increased risk of colorectal cancer (CRC), endometrial cancer (EC), and other cancers, including sebaceous neoplasms [1]. The development of sebaceous neoplasia in Lynch syndrome has historically been considered as a phenotypic variant of Lynch syndrome referred to as Muir-Torre syndrome (MTS). A defining characteristic of cancers that develop in Lynch syndrome is loss of DNA MMR function (MMR-deficiency) evidenced by the loss of MMR protein expression within the tumour.

Sebaceous neoplasms, like CRC and EC, show a high prevalence of MMR-deficiency [2, 3]. Our previous work showed that 31% of the 919 sebaceous neoplasms tested demonstrated MMR-deficiency [2] but this has been reported to be as high as 44% [3]. In CRC and EC, MMR-deficiency can also be caused by somatically acquired mechanisms including biallelic somatic MMR gene mutations (otherwise referred to here as “double somatics”) or *MLH1* promoter hypermethylation [4], the latter being the predominant cause. In sporadic cancers, somatic *MLH1* hypermethylation silences *MLH1*, resulting in loss of MLH1 and PMS2 protein expression i.e. MLH1/PMS2-deficiency. An additional mechanism causing MLH1/PMS2-deficiency is constitutional monoallelic *MLH1* gene promoter hypermethylation (*MLH1* epimutation), a form of Lynch syndrome, which has been shown to be rare. In *MLH1* epimutation cases, one *MLH1* allele is inactivated by hypermethylation with somatic inactivation of the wildtype allele (“second hit”) required to cause MLH1/PMS2-deficiency. *MLH1* epimutation increases the risk of Lynch-spectrum cancers [5], but the prevalence and importance of this mechanism and the somatic causes of MMR-deficiency in sebaceous neoplasm is poorly understood.

This study aims to determine the mechanisms underlying MLH1/PMS2-deficiency in sebaceous neoplasia. Determining the frequency of germline and somatic mechanisms inactivating *MLH1* in sebaceous neoplasms will improve triaging approaches for Lynch syndrome that avoid unnecessary testing and diagnostic delays in people with sebaceous neoplasms.

## Methods

### Study participants and tissue samples

People who developed one or more sebaceous neoplasms at any age were recruited to the Muir-Torre syndrome Sebaceous (MTS) study (Human Research Ethics Committee approval#1648355) from a private pathology practice, Sullivan Nicolaides Pathology [2], Family Cancer Clinics from across Australia and from the Australasian Colon Cancer Family Registry (ACCFR) [4, 6], irrespective of personal or family cancer history, or tumour MMR status. All sebaceous neoplasms underwent immunohistochemical (IHC) testing for expression of the four MMR proteins as part of routine diagnostic testing or as part of the research study as previously described [2, 4]. A total of 28 participants were identified to have a sebaceous neoplasm demonstrating MLH1/PMS2 protein loss, indicative of a defect in the *MLH1* gene. Blood-derived genomic DNA was available for all 28 study participants while macrodissected formalin-fixed paraffin-embedded (FFPE) sebaceous neoplasia tissue DNA was available for 10/28 participants. Seven of the participants including two *MLH1* epimutation carriers have been described previously [4].

## Germline and tumour multigene panel sequencing

Blood-derived DNA samples from all 28 participants underwent multigene panel sequencing of the MMR genes using the amplicon-based target enrichment methodology (Hi-Plex, *n* = 17) or targeted multigene panel sequencing (*n* = 11). Multiplex ligation-dependent probe amplification testing was performed to detect large deletions and duplications. Identified germline pathogenic variants were confirmed using Sanger sequencing. Tumour sequencing was performed on sebaceous neoplasms and matched blood-derived DNA samples from seven participants using a custom targeted multigene panel as previously described [4]. The bioinformatics processing was performed as previously described [4]. Loss-of-heterozygosity was determined using the “*LOHdeTerminator*” (http://github.com/supernifty/LOHdeTerminator, last accessed on 27th of February, 2025) as previously described [4].

### *MLH1* promoter methylation testing

*MLH1* promoter methylation testing of non-Lynch syndrome cases without a germline pathogenic variant in the *MLH1* gene was performed on sebaceous neoplasm tissue DNA (*n* = 10) and blood-derived DNA (*n* = 17) using Methylight with cases positive for *MLH1* promoter methylation undergoing confirmation testing using methylation-sensitive high resolution melting (MS-HRM) as previously described [4, 7].

## Results

The characteristics of the 28 participants with a MLH1/PMS2-deficient sebaceous skin lesion included in the study are shown in Table 1. A germline pathogenic variant in the *MLH1* gene was identified in 11/28 participants and described in Supplementary Table 1. All variants were classified as pathogenic/likely pathogenic by ClinVar or the International Society for Gastrointestinal Hereditary Tumours (InSiGHT) variant databases or both (last accessed on 27th of February, 2025). None of the 28 cases had a variant of unknown significance in *MLH1* or germline pathogenic variant in the *PMS2* gene or the *MSH2* or *MSH6* genes. The 11 cases with *MLH1* pathogenic variants were excluded from further molecular testing as the cause of MLH1/PMS2-deficiency was explained.

**Table 1:** Patient and histological characteristics of the 28 participants diagnosed with sebaceous neoplasms demonstrating loss of MLH1/PMS2 expression included in this study

Characteristics	Individuals (*n* = 28)
Gender, n(%)	
Male	19 (68%)
Female	9 (32%)
Age at diagnosis (years)	
Mean (IQR)	62.4 (15.8)
Range	42–86
Sebaceous lesion type, n (%)	
Sebaceous carcinoma	4 (14%)
Sebaceous adenoma	20 (72%)
Sebaceoma	4 (14%)
Sebaceous lesion location, n (%)	
Head/neck	18 (64%)
Trunk/limbs	10 (36%)

**Fig. 1:**
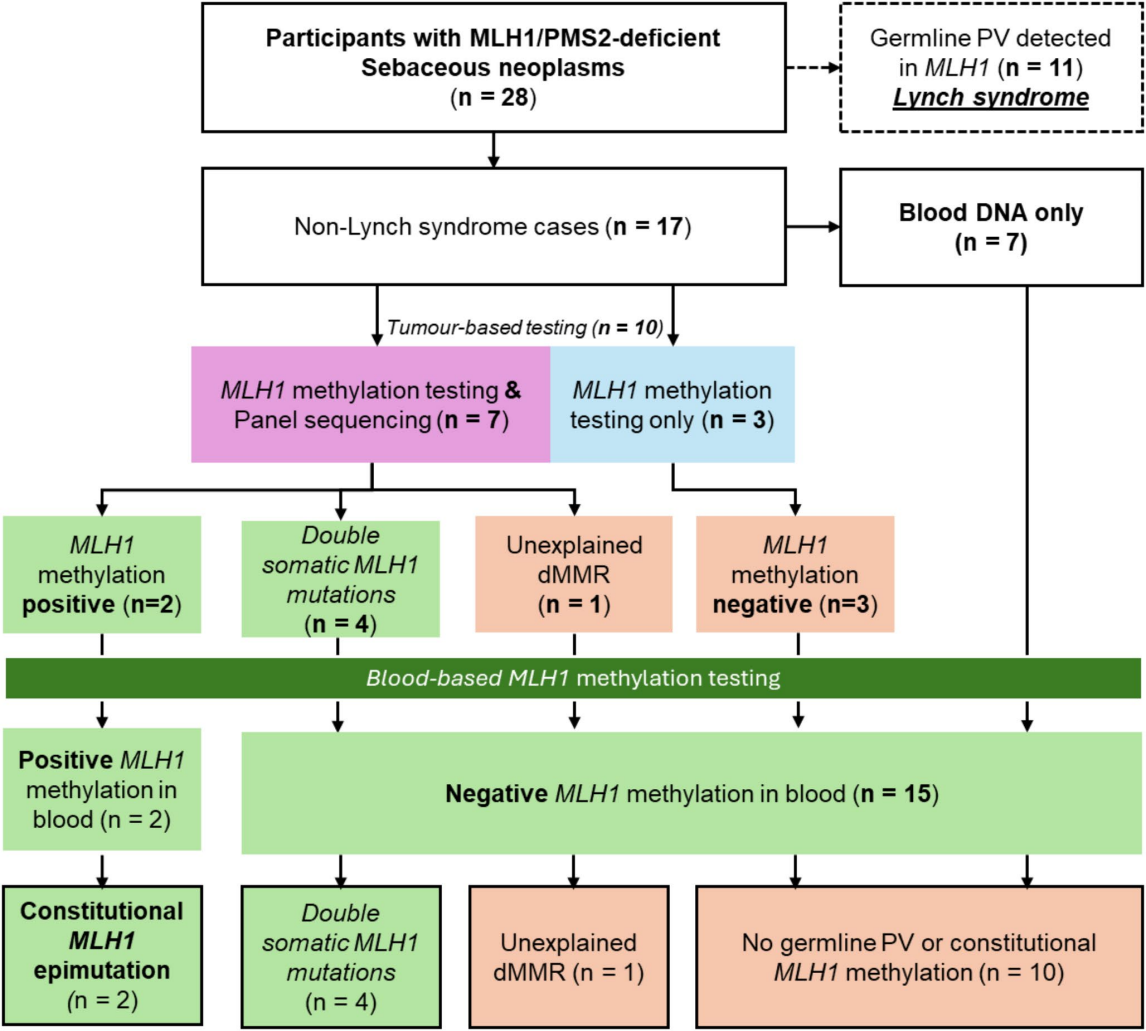
Flow diagram showing the germline and somatic testing approaches and outcomes from each test applied in this study

Of the remaining 17 cases without a germline *MLH1* pathogenic variant, sebaceous neoplasia tissue DNA was available for testing for 10/17 participants. Tumour-based *MLH1* methylation testing was performed on all 10 sebaceous neoplasms identifying two *MLH1* methylation positive samples, which were cross-validated by both MethyLight and MS-HRM assays (P-012 and P-019). Tumour sequencing was performed only on 7/10 sebaceous neoplasia samples as three cases had insufficient tissue DNA remaining after *MLH1* methylation testing (Fig. 1). Tumour sequencing identified two somatic mutations in the *MLH1* gene (i.e. double somatic *MLH1* mutations) in 4/7 sebaceous neoplasms (Fig. 1).

To determine if the two somatic *MLH1* hypermethylation positive cases, P-012 & P-019, were related to constitutional *MLH1* epimutation, blood-based *MLH1* methylation testing was performed. Both cases were positive for *MLH1* hypermethylation in blood DNA, indicating constitutional *MLH1* epimutation and not somatic *MLH1* hypermethylation (Fig. 1). For completeness, the remaining 8/10 cases that were negative for somatic *MLH1* methylation were also tested for constitutional *MLH1* epimutation with none found positive for *MLH1* hypermethylation in blood-derived DNA. To identify additional *MLH1* epimutation cases, blood-based *MLH1* methylation testing was performed on the seven non-Lynch syndrome cases without sufficient tumour DNA but no additional *MLH1* epimutation cases were identified (Fig. 1). These findings indicated that constitutional rather than somatic *MLH1* hypermethylation was the mechanism underlying MLH1/PMS2-deficiency in sebaceous neoplasia.

One sebaceous neoplasm underwent all tumour- and blood-based testing but no evidence of a germline *MLH1* pathogenic variant, somatic *MLH1* hypermethylation or *MLH1* epimutation, or double somatic *MLH1* mutations was identified, therefore, the cause of MLH1/PMS2-deficiency in this case was considered unexplained (Fig. 1). For the seven non-Lynch syndrome cases where only blood-based testing was completed, no constitutional *MLH1* epimutation was identified, suggesting that a somatic aetiology likely explains the MLH1/PMS2-deficiency in these cases but could not be confirmed due to insufficient tissue DNA (Fig. 1).

Table 2 shows the clinico-molecular characteristics of the two cases identified with *MLH1* epimutations. P-012 developed a colon cancer at 55 years and sebaceous adenoma at 61 years of age, with both tumours demonstrating MLH1/PMS2-deficiency and positivity for *MLH1* hypermethylation. The participant had no first or second degree relatives with CRC, EC or sebaceous neoplasms. The targeted multigene panel sequencing identified loss-of-heterozygosity across the *MLH1* gene in the colon cancer as the “second hit” but a second hit in the sebaceous adenoma was not identified. For the second *MLH1* epimutation case P-019, the sebaceous adenoma was diagnosed at 49 years of age and showed loss-of-heterozygosity of the wildtype allele across the *MLH1* gene. No cancer was reported in this participant at recruitment. The participant had no first or second degree relatives with CRC, EC, or sebaceous neoplasms. For both *MLH1* epimutation cases, no germline pathogenic variants were identified in the *MLH1* promoter region that may have contributed to the constitutional methylation, meaning these two cases would be considered primary *MLH1* epimutations and not secondary to a germline promoter variant. The mean diagnosis age of two *MLH1* epimutation carriers was not significantly different from double somatic (*P =* 0.47) or from MTS/Lynch syndrome (*P =* 0.36) (Supplementary Table 2).

**Table 2:** Characteristics of two participants identified with constitutional *MLH1* epimutation. ^1^*MLH1* methylation level in DNA derived from sebaceous neoplasms detected using Methylight. ^2^*MLH1* methylation level in blood-derived DNA detected using Methylight. ^3^ the custom-designed panel sequencing covers up to 1500 bp upstream from the *MLH1* transcription start site. LOH - loss-of-heterozygosity ^4^ presence of Lynch-spectrum cancers (e.g. CRC, EC, sebaceous neoplasms) in first- and/or second-degree relatives

Patient_ID	Sex	Neoplasia (diagnosis age)	Anatomical location	MMR protein expression	*MLH1* promoter methylation (%)^1^	Somatic mutation in *MLH1*	*MLH1* promoter methylation in blood DNA (%)^2^	Germline *MLH1* promoter variants^3^	Type of *MLH1* epimutation	Family history of cancer^4^
P-012	Female	Sebaceous adenoma (61y)	Left anterior neck	MLH1/PMS2 loss	Positive (20%)	Not detected	Positive (32%)	None identified	Constitutional *MLH1* epimutation (primary)	No
		CRC (55y)	Caecum	MLH1/PMS2 loss	Positive (75%)	LOH				
P-019	Male	Sebaceous adenoma (49y)	Chest	MLH1/PMS2 loss	Positive (58%)	LOH	Positive (3%)	None identified	Constitutional mosaic *MLH1* epimutation (primary)	No

## Discussion

This study investigated germline and somatic mechanisms underlying MLH1/PMS2-deficiency in sebaceous neoplasms. After identifying Lynch syndrome cases due to germline *MLH1* pathogenic variants, double somatic *MLH1* mutations (*n* = 4) was the main cause, followed by Lynch syndrome due to constitutional *MLH1* epimutations (*n* = 2). A single case with sufficient tissue DNA enabling both *MLH1* methylation and *MLH1* somatic mutation testing did not identify either as a mechanism, potentially suggesting a false-positive MLH1/PMS2-deficiency or other mechanisms not captured by the techniques used in this study (e.g. large structural variants, deep-intronic variants).

Three constitutional *MLH1* epimutation cases with sebaceous neoplasms have been described to date [4, 8]. Zyla et al. reported a female patient diagnosed with CRC (33yrs), squamous cell carcinoma of the vulva (37yrs), a facial sebaceous adenoma (45yrs) and EC (52yrs) [8]. All tumours exhibited MLH1/PMS2-deficiency and *MLH1* methylation testing of the EC was positive. Subsequent blood-based *MLH1* methylation testing identified constitutional *MLH1* epimutation, explaining development of multiple MLH1-deficient cancers. In our previous study, we investigated the mechanisms underlying MMR-deficiency in CRC, EC and sebaceous neoplasms from people with “suspected Lynch syndrome” or “Lynch-like syndrome” [4]. *MLH1* methylation analysis identified two index *MLH1* epimutation cases, both with sebaceous neoplasia [4] and are described in this study. This present study, the first to examine both somatic *MLH1* methylation and constitutional *MLH1* epimutation in sebaceous neoplasms, has provided an important extension of this work demonstrating that somatically acquired *MLH1* hypermethylation was not present in the 10 sebaceous neoplasia cases tested, suggesting that this is not a common cause for MLH1/PMS2 MMR-deficiency unlike in CRC and EC.

The tumourigenic mechanism in constitutional *MLH1* epimutation carriers is intrinsically different from somatic *MLH1* methylation. In CRCs and ECs, somatic *MLH1* methylation causes biallelic hypermethylation of the *MLH1* gene promoter resulting in complete silencing of the gene [5]. Somatic *MLH1* hypermethylation is specific to the malignant cells and associated with older age at diagnosis [9]. In constitutional *MLH1* epimutation, monoallelic *MLH1* hypermethylation inactivates one allele and a second somatic hit causes biallelic silencing of *MLH1* resulting in loss of DNA MMR function [5]. As the tumourigenic process in *MLH1* epimutation follows the same process as Lynch syndrome resulting from germline MMR pathogenic variants, cancer diagnosis age in *MLH1* epimutation is thought to be similar to Lynch syndrome and younger than cases with somatic *MLH1* hypermethylation. Therefore, triaging patients for constitutional *MLH1* epimutation testing has often been guided by a younger age at diagnosis [9]. The diagnosis ages of sebaceous neoplasms in three *MLH1* epimutation cases, including two from this study and one from Zyla et al. [8], were 55, 49 and 45 years. This is seemingly younger than the mean age of 68 years presented by our previous study of 882 people with sebaceous neoplasia [2], and may suggest that younger age at diagnosis could be useful for differentiating *MLH1* epimutation carriers from sporadic cases.

This study demonstrates the importance of both germline and tumour testing in individuals diagnosed with MLH1/PMS2-deficient sebaceous neoplasms. Based on our findings, a potential testing strategy for people with MLH1/PMS2-deficient sebaceous neoplasms would be initial germline MMR gene testing to identify a *MLH1* pathogenic variant coupled with *MLH1* epimutation testing (Supplementary Fig. 1). For those without germline MMR pathogenic variant, tumour-based testing to identify biallelic somatic *MLH1* mutations would provide a definitive diagnosis for an important proportion of MLH1/PMS2-deficient cases. Differentiating constitutional *MLH1* epimutation from somatic *MLH1* hypermethylation is clinically important, as having Lynch syndrome related to*MLH1* epimutation is associated with an increased risk of developing multiple cancers including CRC and EC and may inform familial risk assessment [5]. Furthermore, differentiating primary *MLH1* epimutations from secondary *MLH1* epimutations resulting from a germline *MLH1* promoter pathogenic variant is important for risk assessment in relatives. The two cases identified in our study were primary epimutation cases based on the testing undertaken.

A limitation of this study was the inability to complete sebaceous tissue testing in 10 cases due to the small lesion sizes resulting in insufficient DNA for testing. Although Lynch syndrome related to germline pathogenic variant and constitutional *MLH1* epimutation were confidently excluded in these cases, somatic *MLH1* methylation cannot be ruled out in these 10 cases. However, findings from the sebaceous tumours we could test suggest biallelic somatic *MLH1* mutations are a more likely cause of MLH1/PMS2-deficiency than somatic *MLH1* hypermethylation. Validation of our findings will help guide clinical diagnostic testing for people with MLH1/PMS2-deficient sebaceous neoplasia. With a sample size of 28 cases, this study is the largest to perform systematic investigation of germline and somatic mechanisms of MLH1/PMS2-deficient sebaceous neoplasms. We identified 11 participants with *MLH1 * pathogenic variants, which was the most prevalent mechanism for MLH1/PMS-deficiency, however, this could be an over-representation due to a potential ascertainment bias resulting from recruitment of our some of our participants from Family Cancer Clinics and the ACCFR.

## Conclusions

This study found no evidence of somatic *MLH1* hypermethylation in the MLH1/PMS2-deficient sebaceous neoplasms tested and highlights Lynch syndrome due to constitutional *MLH1* epimutation is a rare but important cause. *MLH1* epimutation carriers are predisposed to multiple cancer so their identification is clinically important to manage their cancer risk.. This study suggests that somatic *MLH1* hypermethylation is not a common mechanism in sebaceous neoplasia, which contrasts their predominance in CRC and EC and indicates a different triaging approach should be considered for people with MLH1/PMS2-deficient sebaceous neoplasia potentially starting with germline investigations.

## Electronic supplementary material

Below is the link to the electronic supplementary material.

Supplementary Material 1

Supplementary Material 2

## Data Availability

The data that support the findings of this study are available from the corresponding author upon reasonable request.
