# Supplementary material for: Causes of DNA mismatch repair deficiency in sebaceous skin lesions demonstrating loss of MLH1 protein expression: constitutional over somatic MLH1 promoter methylation
Source: Fam Cancer. 2025 Apr 10;24(2):36. doi: 10.1007/s10689-025-00456-w (PMC11985684; doi:10.1007/s10689-025-00456-w)
Supplement: Supplementary file 1 — Supplementary Material 1 [file 10689_2025_456_MOESM1_ESM.pdf]

Supplementary Table 1

MTS_ID	Sex	Sebaceous Neoplasm (age at diagnosis, years)	Anatomical location	Other cancers (age at diagnosis, years)	Multiple sebaceous lesions	Germline *MLH1* variant	ClinVar pathogenicity
P-013	Female	Sebaceous adenoma (57)	Post-auricular back of skull	Not identified	No	c.350C>T (p.Thr117Met)	Pathogenic
P-018	Male	Sebaceous adenoma (49)	Anterior chest	Not identified	No	c.1038+1G>T	Pathogenic/Likely pathogenic
P-020	Male	Sebaceous adenoma (70)	Right cheek	CRC (66)	No	c.1975C>T (p.Arg659Ter)	Pathogenic
P-021	Male	Sebaceous adenoma (62)	Right lower back	CRC (48)	2 sebaceous lesions (42 and 62y)	c.588+1G>T	Pathogenic
P-022	Female	Sebaceous adenoma (42)	Left upper thigh	CRC (39), Stomach (42)	9 sebaceous lesions between age of 42-50y	c.1713_1716delTGGT (p.Phe571LeufsTer19)	Pathogenic (InSiGHT)
P-023	Male	Sebaceous adenoma (53)	Left flank	Not identified	No	c.1683C>G (p.Tyr561Ter)	Pathogenic
P-024	Female	Sebaceous adenoma (60)	Nose	CRC (46)	No	c.1554dupT (p.Glu519fs)	Pathogenic
P-025	Female	Sebaceous adenoma (74)	Right chest	Not identified	8 sebaceous lesions between age of 74-86y	c.380+1G>A r.spl? p.?	Likely pathogenic
P-026	Male	Sebaceoma (68)	Left axillary line	CRC (56)	No	c.1713_1716delTGGT (p.Phe571LeufsTer19)	Pathogenic (InSiGHT)
P-027	Male	Sebaceous adenoma (76)	Left nasal tip	CRC (39), Small intestine (72)	2 sebaceous lesions (both at 76y)	c.199G>A (p.Gly67Arg)	Pathogenic
P-028	Male	Sebaceous adenoma (74)	Left medial canthus	Not identified	No	c.1713_1716delTGGT (p.Phe571LeufsTer19)	Pathogenic (InSiGHT)

Supplementary Table 2

Characteristics	MTS (germline *MLH1* PV)	Constitutional *MLH1* epimutation	Double somatic *MLH1* mutations	Unexplained cause of MLH1/PMS2 loss
Gender, n (%)				
Male	7 (64%)	1 (50%)	4 (100%)	7 (64%)
Female	4 (36%)	1 (50%)	0	4 (36%)
Age at diagnosis (years)				
Mean (IQR)	63.7 (17.1)	55.5 (na)	62.4 (16.8)	62.3 (12.2)
Sebaceous lesion type, n (%)				
Sebaceous adenoma	8 (73%)	2 (100%)	3 (75%)	7 (64%)
Sebaceous carcinoma	2 (18%)	0	1 (25%)	1 (9%)
Sebaceoma	1 (9%)	0	0	3 (27%)
Location of sebaceous lesion, n (%)				
Head/neck	5 (45%)	1 (50%)	**4 (100%)**	8 (73%)
Trunk/limb	6 (55%)	1 (50%)	0	3 (27%)

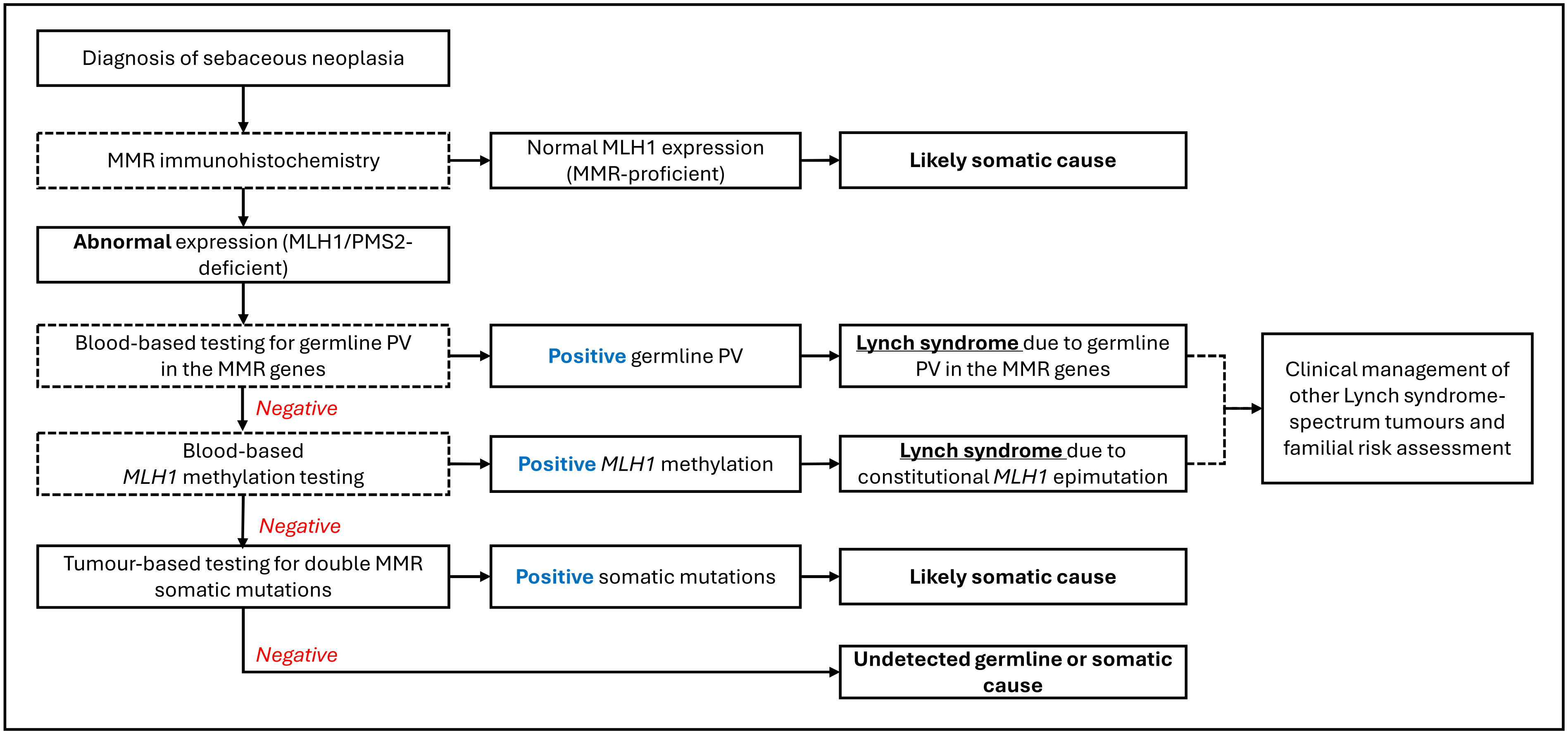

Supplementary Figure 1
