# Supplementary material for: Causes of DNA mismatch repair deficiency in sebaceous skin lesions demonstrating loss of MLH1 protein expression: constitutional over somatic MLH1 promoter methylation
Source: Fam Cancer. 2025 Apr 10;24(2):36. doi: 10.1007/s10689-025-00456-w (PMC11985684; doi:10.1007/s10689-025-00456-w)
Supplement: Supplementary file 2 — Supplementary Material 2 [file 10689_2025_456_MOESM2_ESM.docx]

***Supplementary Table and Figure legends***

**Supplementary Table 1.** Characteristics of the 11 participants identified to have a germline pathogenic variant in the *MLH1* gene (NM_*000249.4*).

**Supplementary Table 2.** Summary of the 28 participants by the cause of MLH1/PMS2-deficiency including Lynch syndrome (n = 11), constitutional *MLH1* epimutation (n = 2), double *MLH1* somatic mutations (n = 4) and unexplained cases (n = 11).

**Supplementary Figure 1**. Flow diagram showing the proposed algorithm for triaging people with MLH1/PMS2-deficient sebaceous neoplasia to determine the germline and somatic causes.
